# Network Meta-analysis Comparing Efficacy, Safety and Tolerability of Anti-PD-1/PD-L1 Antibodies in Solid Cancers

**DOI:** 10.7150/jca.57413

**Published:** 2021-05-19

**Authors:** Laith Al-Showbaki, Michelle B. Nadler, Alexandra Desnoyers, Fahad A. Almugbel, David W. Cescon, Eitan Amir

**Affiliations:** Department of Medical Oncology and Hematology, Princess Margaret Cancer Center, University Health Network, Faculty of Medicine, University of Toronto, Toronto, Canada.

**Keywords:** Immunotherapy, Programmed Cell Death 1 Receptor, Review, Clinical Trial, Phase III as Topic

## Abstract

**Background:** Multiple anti-PD-1/PD-L1 antibodies have been approved, and in some diseases, there is a choice of more than one. Comparative efficacy, safety and tolerability are unknown.

**Methods:** Randomized trials (RCTs) supporting the registration of single agent anti-PD1 or anti-PDL1 inhibitors between 2015-2019 were identified. We extracted the hazard ratio (HR) for overall survival (OS) and calculated the odds ratio (OR) for commonly reported safety and tolerability outcomes. We then performed a network meta-analysis, reporting multiple pair-wise comparisons between different anti-PD-1/PD-L1 antibodies.

**Results:** Sixteen RCTs comprising 10673 patients were included; 10 in non-small-cell lung cancer, 2 in melanoma, 2 in head and neck squamous cell carcinoma and 2 in urothelial cancer. Compared to pembrolizumab, efficacy was similar for nivolumab (HR: 1.02 95% CI: 0.91-1.14) and for atezolizumab (HR: 0.97 95% CI: 0.85-1.10), however, avelumab appeared inferior (HR: 1.30, 95% CI: 1.06-1.56). Pembrolizumab showed similar odds of serious adverse events (SAEs) as nivolumab (OR: 1.12, 95% CI: 0.56-2.27) and atezolizumab (OR: 1.05, 95% CI: 0.55-2.04). Compared to nivolumab, atezolizumab was associated with more SAEs (OR: 2.14, 95% CI: 1.47-3.12). Avelumab had the lowest odds of grade 3-4 adverse events compared to pembrolizumab (OR: 0.42, 95% CI: 0.24-0.74), nivolumab (OR: 0.38, 95% CI: 0.24-0.62) and atezolizumab (OR: 0.21, 95% CI: 0.14-0.33). The odds of treatment discontinuation without progression were similar between nivolumab and atezolizumab (OR: 1.20, 95% CI: 0.73-2.00), and between pembrolizumab and nivolumab (OR: 1.35, 95% CI: 0.83-2.17), but was higher with atezolizumab compared to nivolumab (OR: 2.56, 95% CI: 1.29-5.00). Pembrolizumab was associated with higher OR of immune-related adverse events (IRAEs) compared to nivolumab (OR: 2.12, 95% CI: 1.49-3.03) and atezolizumab (OR: 1.63, 95% CI: 1.09-2.43).

**Conclusions:** Pembrolizumab, nivolumab, and atezolizumab have similar efficacy. Avelumab appears less efficacious. Safety and tolerability seem better with avelumab, but worse with atezolizumab and pembrolizumab.

## Introduction

Over the past decade, numerous randomized controlled trials (RCTs) of immune checkpoint inhibitors (ICIs) have been performed. Compared to standard of care, ICIs, targeting the programed death receptor-1 (PD-1) or its ligand (PD-L1), have been shown to be an efficacious therapeutic tool in several cancer sites. Additionally, these drugs have a generally favourable safety profile as compared to conventional treatment 1. As a result, multiple anti-PD-1/PD-L1 drugs have been approved in cancer and are in routine use.

In some diseases, more than one anti-PD-1/PD-L1 antibody is approved for the same indication allowing clinicians, patients and funders to select one over another. Ideally, such therapeutic decision-making should be based on relative efficacy and toxicity. However, we are not aware of any head-to-head data informing of the differential efficacy safety and tolerability of different anti-PD-1/PD-L1 antibodies. Several meta-analyses have been performed to study differential efficacy and safety of available anti PD1/PD-L1 inhibitors indirectly. Liang et al. compared the efficacy and safety of anti-PD-1/PD-L1 antibodies in non-small cell lung cancer (NSCLC) using objective response rate (ORR) and frequency of adverse events (AEs) 2. Almutairi et al. investigated the difference in overall survival (OS), progression-free survival (PFS) of anti-PD-1/PD-L1 antibodies in NSCLC 3. Data have also been reported in advanced melanoma 4 and in advanced nasopharyngeal carcinoma 5.

Here, we report on a network meta-analysis comparing different anti-PD-1/PD-L1 antibodies, across different disease sites, aiming to provide a more clear understanding of the differential efficacy and toxicity of these agents.

## Methods

### Source of data

Drug@FDA was used to identify FDA approved indications of anti-PD-1/PD-L1 antibodies, up to February 2020, (including pembrolizumab, nivolumab, cemiplimab, atezolizumab, avelumab and durvalumab). RCTs supporting the registration of anti-PD-1/PD-L1 antibodies were identified using a search of MEDLINE (host: PubMed) using the drug name and the approved indications as keywords. RCTs that studied anti-PD-1/PD-L1 antibodies in combination with other therapeutic agents such as chemotherapy, targeted therapy or radiotherapy were excluded, as were trials of anti-cytotoxic-T-lymphocytes-associated protein 4 (CTLA-4) monoclonal antibodies. When duplicates of RCTs were identified, we included the most recent report for both efficacy and safety (noting that these reports may not have been the same). When approval was based on subgroups defined by PDL-1 status, we compared efficacy outcomes in the subgroup for which approval was achieved (if applicable). Safety and tolerability were based on all trial participants exposed to treatment (per protocol analyses). We only included trials where more than one anti-PD-1/PDL-1 antibody were approved for the same indication in one disease site, to initiate a clinically and statistically meaningful head to head efficacy and safety comparisons between at least two anti-PD-1/PDL-1. Pembrolizumab was approved for 15 disease site, in some disease sites it was approved for use in multiple settings (adjuvant and palliative). Nivolumab, atezolizumab, durvalumab and cemiplimab was approved for several disease sites in different indications. However, Advanced NSCLC, both in first line and second line palliative treatment, (pembrolizumab, nivolumab and atezolizumab), metastatic HNSCC as first line palliative treatment (pembrolizumab and nivolumab), Metastatic urothelial cancer, as in second line palliative therapy, (nivolumab and atezolizumab), and metastatic melanoma, as in first line treatment, (pembrolizumab and nivolumab) appeared to be the only indications where, two or more anti-PD-1/PDL-1 antibodies were approved as in monotherapy use. Please see Table-1 for complete list of FDA approved anti-PD-1/PDL-1 antibodies. For example, Although durvalumab was approved as in single agent as a consolidation therapy, following concurrent platinum-based chemotherapy and radiation therapy, only in stage III NSCLC, we did not include durvalumab in the comparators due to the significant difference in disease stage and treatment structure, compared to other approved anti-PD-1/PDL-1 antibodies in the same disease site. In addition, while both pembrolizumab and nivolumab were both approved in advanced SCLC, as in single agent palliative therapy, the variation in the previous treatment exposure, pembrolizumab is approved as a second line palliative therapy while nivolumab is approved as a third line palliative therapy, could have affected the efficacy and safety comparisons, we did not compare pembrolizumab and nivolumab in advanced SCLC. On the other hand, although avelumab was not approved in the treatment of advanced NSCLC, we included the differential efficacy results of avelumab compared to other anti-PD-1/PDl-1 antibodies in second line palliative treatment of advanced NCSCL, to highlight the distinctive avelumab efficacy and safety outcome.

### Data collection

Data collection was performed between October 2019 and January 2020. One author (LA-S) reviewed the publication of each RCT and extracted the following data. For efficacy, we extracted the hazard ratio (HR) for overall survival (OS) as well as the 95% confidence intervals (CI). Safety and tolerability data comprised the proportion of participants exposed to treatment who were observed to have any grade adverse events (AEs) as well as a subgroup with grade 3 or 4 treatment related AEs, serious adverse events (SAEs), immune-related adverse events (IRAEs), and events leading to discontinuation of the drug without disease progression or death.

### Data synthesis and statistical analysis

For each safety and tolerability outcome, we calculated the odds ratio (OR) and respective standard error (SE) and/or 95% confidence intervals (CI). ORs were calculated by the Mantel Haenszel method using Review Manager Version 5.3 (The Cochrane Collaboration, Copenhagen, Denmark). Data were pooled only in disease sites where more than one anti-PD-1/PD-L1 antibody was approved. Pair-wise comparisons were performed to explore differential efficacy (as measured by the HR for OS) and safety and tolerability (as measured by the OR for each safety and tolerability measure described above). When data were available from more than one pair-wise comparison, they were pooled using generic inverse variance. Due to the substantial clinical heterogeneity, analyses were performed using random effects modeling irrespective of statistical heterogeneity. Network meta-analysis was performed using WINBUGS within Microsoft Excel (Microsoft Corp, Redmond WA). Statistical tests were two-sided, and statistical significance was defined as p < 0.05. No correction was made for multiple statistical testing.

## Results

A total of 16 RCTs 6-21 comprising 10673 patients were included in the analysis. Of these, ten were in NSCLC, two in advanced melanoma, two in head and neck squamous cell carcinoma and two in metastatic urothelial cancers. Of the included trials, there were two phase II RCTs, while the rest comprised phase III RCTs. Eight trials (50%) involved 1^st^ line treatment. These trials resulted in a total of 26 different pair-wise comparisons of different ICIs. Characteristics of included studies are shown in Table 1.

### Efficacy

**Figure 1:**
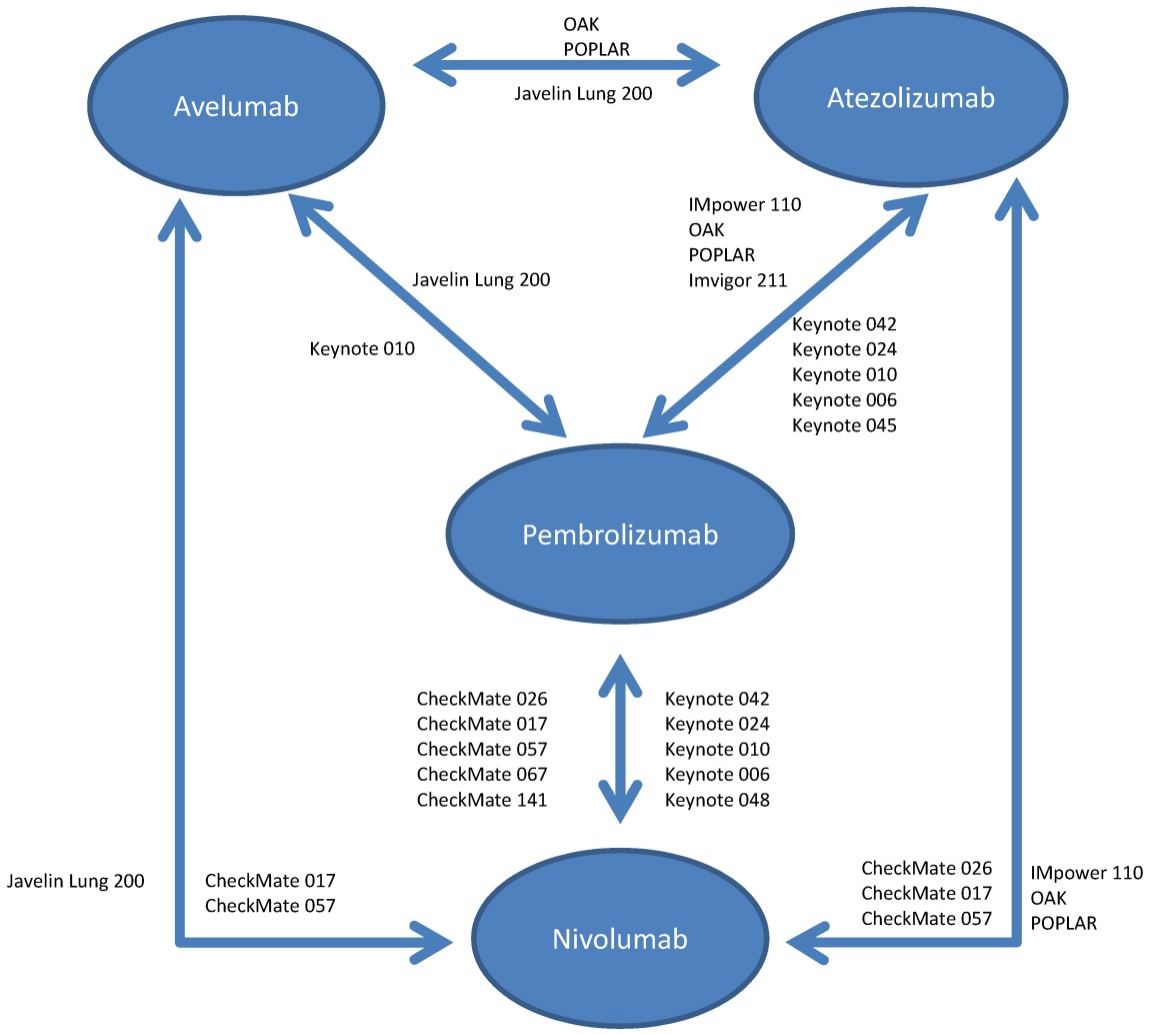
Schema for comparisons included in the network meta-analysis.

The available RCTs permitted nine indirect pair-wise comparisons between pembrolizumab and nivolumab, six comparisons between pembrolizumab and atezolizumab, two comparisons between pembrolizumab and avelumab, five comparisons between nivolumab and atezolizumab, two comparisons between nivolumab and avelumab and two comparisons between atezolizumab and avelumab (see Figure 1). Results of individual pair-wise comparisons are shown in Table 2. Data reporting pooled efficacy data are shown in Table 3.

Compared to pembrolizumab, efficacy was similar for nivolumab and for atezolizumab. However, avelumab appeared inferior. Nivolumab and atezolizumab showed similar efficacy, while avelumab was associated with lesser efficacy compared to nivolumab and atezolizumab.

### Safety and tolerability

The results of all safety and tolerability data are shown in Supplementary Table 1. The OR for treatment-related adverse events was similar for pembrolizumab, when compared to nivolumab (OR for any AE: 0.89, 95% CI: 0.63-1.27 and OR for grade 3/4 AEs: 1.12, 95% CI: 0.86-1.47). Pembrolizumab had lower odds of adverse events compared to atezolizumab (OR for any AE: 0.54, 95% CI 0.37-0.78). However, the OR for grade 3/4 AEs was comparable between both agents (OR of grade 3/4 AEs: 0.84, 95%: 0.64-1.10). Avelumab was associated with similar odds for adverse events of any grade when compared to pembrolizumab, although there were lower odds of higher grade adverse events (OR for any AE: 0.73, 95% CI: 0.44-1.22 and OR for grade 3/4 AEs: 0.42, 95% CI: 0.24-0.74). Compared to atezolizumab, avelumab was associated with lower odds of all adverse events (OR for any AE: 0.43, 95% CI: 0.23-0.78 and OR for grade 3/4 AEs: 0.21, 95% CI: 0.14-0.33). The OR of AEs was similar between avelumab and nivolumab (OR for any AEs: 0.92, 95% CI: 0.49 to 1.72), while the risk of grade 3/4 AEs was lower with avelumab (OR for grade 3/4 AEs: 0.38, 95% CI: 0.24-0.62). Compared to nivolumab atezolizumab was associated with more adverse events (OR for any AE: 2.31, 95%: 1.34-3.94 and OR for grade 3/4 AEs: 1.84, 95% CI: 1.37-2.47).

Pembrolizumab had similar odds of SAEs compared to nivolumab (1.12, 95% CI: 0.56-2.24) and atezolizumab (1.05, 95% CI: 0.55-2.00). Atezolizumab was associated with higher odds of SAEs compared to nivolumab (OR: 2.14, 95% CI: 1.47-3.12).

Compared to pembrolizumab, both nivolumab (OR: 0.47, 95% CI: 0.33-67) and atezolizumab (OR: 0.61, 95% CI: 0.41-0.91) were associated with fewer IRAEs. Additionally, compared to pembrolizumab, treatment discontinuation was similar with nivolumab (OR: 0.74, 95% CI: 0.47-1.20), but lower with atezolizumab (0.39, 95% CI: 0.20-0.77). The rates of IRAEs and treatment discontinuation were similar for atezolizumab, compared to nivolumab (OR: 1.14, 95% CI: 0.60-2.16 and OR: 0.83, 95% CI: 0.50-1.36, respectively).

## Discussion

In this study, we performed a network meta-analysis to compare the differential efficacy, safety and tolerability of different anti-PD-1/PD-L1 antibodies as evaluated in randomized monotherapy trials in advanced malignancies. No statistically significant difference in efficacy was observed between pembrolizumab, nivolumab and atezolizumab. However, avelumab appeared less efficacious. In contrast, avelumab appeared to be associated with the most favourable safety and tolerability profile compared to other anti-PD-1/PD-L1 antibodies. Nivolumab was as effective as pembrolizumab and atezolizumab but seemed to have less toxicity in general. As multiple anti-PD-1/PD-L1 antibodies are approved in a number of disease sites, these data can help in clinical decision making.

Compared to other analyses aiming to compare different anti-PD-1/PD-L1 antibodies in individual disease sites 1-5, our analysis comprised a much larger number of studies and therefore, may be generalized more widely. We also explored more definitive efficacy outcomes (OS) as well as exploring a breadth of safety and tolerability outcome measures. We excluded RCTs of anti-PD-1/PD-L1 antibodies in combination therapy in order to eliminate the potential effect of the companion therapy on efficacy and toxicity. The result of this however, is that our data apply only to the use of single agent anti-PD-1/PD-L1 inhibitors.

The different anti-PD-1/PD-L1 antibodies have distinct properties. For pembrolizumab and nivolumab, there are significant overlaps between the epitopes of both antibodies and the PD-1 ligand binding site 22. The interaction of pembrolizumab with PD-1 is primarily dependent on the C′D loop of free PD-1. In addition, pembrolizumab interacts with the C and C′ strands of PD-1, ensuring that it competes with the binding of PD-1 ligands. Nivolumab binds PD-1 mainly through the *N*-terminal extension, which is located outside the immunoglobulin-like V-type domain of PD-1 and is not involved in ligand recognition 23. As such, the proposed mechanism of action of pembrolizumab and nivolumab is singular: blockade of the interaction between PD-1 and its ligands through competitive binding of PD-1 24, 25. The limited difference in the clinical efficacy between nivolumab and pembrolizumab is likely explained by the minimal differences in the specific interactions of their epitopes.

Atezolizumab and avelumab target the PD-L1 ligand rather than the PD-1 receptor. The Fab fragments of atezolizumab bind close to the *N*-terminus of PD-L1. In contrast, avelumab binds more perpendicularly to the face of PD-L1. Despite the different binding orientations and different epitopes between antibodies, they all interact with the five hotspot residues (Y56, E58, R113, M115, and Y123) on the central CC'FG β sheet within PD-L1 26-29 while the distinctive mechanism of action and resultant inhibition in the T-cell signaling is expected to be unrelated to the method of PD-1 blockade, our data show modest differences in efficacy, safety and tolerability between avelumab and the other anti-PD-1/PD-L1 inhibitors. However, differences in study design and patient populations, may have contributed to the observed effects, at least for anti-PD-L1 antibodies if not anti-PD-1 antibodies. Some studies selected patients based on PD-L1 expression. There was also heterogeneity in the biomarker assay utilized and threshold used to identify positive expression. Since efficacy is a direct product of the predictive biomarker, it's possible that the differences observed may be influenced by differences in patient selection.

This study has limitations. First, our data are based on a relatively small number of included RCTs. Additionally, the higher proportion of studies in some disease sites such as NSCLC means that results may be influenced more by this disease site. Second, not all safety and tolerability outcome measures were available for each trial. Third, we extracted study summary-level data as we did not have access to more granular individual patient data. Fourth, there was substantial inter-study heterogeneity in patient population (disease type, line of therapy, eligibility criteria etc.) and this may have had an impact on safety and efficacy. For example, in metastatic head and neck squamous cell carcinoma, the outcomes in the control group were different among included trials (median OS in KEYNOTE-048 10.7 months in total population, and 10.3 months in CPS>1% compared to 5.1 months in total population and 4.6 months in CPS>1% group in Checkmate-141). Additionally, some variation was noted in the different control group regimens in Checkmate-141 (docetaxel: 5.8 months, methotrexate: 4.6 months and cetuximab 4.1 months). However, such differences were not observed with all disease sites with variable control group therapy. For example, in metastatic urothelial carcinoma, despite differences in control group therapy, included trials had similar control group outcomes (median OS in KEYNOTE-045 7.4 months and in IMvigor211 8.6 months).

In our study, all the included trial involved treatment in the palliative settings in several advanced disease sites, which did emphasize any head to head comparisons between different anti-PD-1/PDL-1 in early stage malignancies. Finally, anti-PD-1/PD-L1 antibodies are used in a wide variety of disease sites which were not included in our analysis. This was due either to anti-PD-1/PD-L1 antibodies being approved based on single-arm rather than randomized studies, because multiple RCTs of different anti-PD-1/PD-L1 antibodies were not available or that anti-PD-1/PD-L1 antibodies were used in combination with other drugs and not as monotherapy. This precluded inclusion in a network meta-analysis. As such, these data may not be applicable to disease sites such as classical Hodgkin lymphoma, primary mediastinal large B-cell lymphoma, microsatellite instability-high cancers, gastro-esophageal cancers, hepatocellular carcinoma among others.

In summary, pembrolizumab, nivolumab, and atezolizumab have similar efficacy, but avelumab appears less efficacious. Safety and tolerability seem better with avelumab, but worse with atezolizumab and pembrolizumab. These data provide important information for the trade-offs between benefits and risks on anti-PD-1/PD-L1 inhibitors.

## Supplementary Material

Supplementary table S1.

## Figures and Tables

**Table 1:** Characteristics of included randomized controlled trials

Trial	Year	Experimental Arm	Method of PD-l testing	Treatment line	Disease site	Control Arm	Sample size
Keynote-042	2019	Pembrolizumab 200 mg every 3 week	PD-L1 IHC 22C3 pharmDx assay	1st line	NSCLC	Platinum based combination therapy	1274
Keynote-024	2016	Pembrolizumab 200 mg every 3 weeks	PD-L1 IHC 22C3 pharmDx assay	1st line	NSCLC	Platinum based combination therapy	305
Keynote-010	2015	Pembrolizumab 2 mg/kg every 3 weeks(1.1)	PD-L1 IHC 22C3 pharmDx assay	2nd line	NSCLC	Docetaxel	1034
CheckMate-057	2015	Nivolumab 3 mg/kg every 2 weeks	Rabbit 28-8 (Dako)	2nd line	NSCLC (Non-Squamous)	Docetaxel	582
CheckMate-017	2015	Nivolumab 3 mg/kg every 2 weeks	Rabbit 28-8 (Dako)	2nd line	NSCLC (Squamous)	Docetaxel	272
CheckMate-026	2017	Nivolumab 3 mg/kg every 2 weeks	Rabbit 28-8 (Dako)	1st line	NSCLC	Platinum based combination therapy	541
IMpower110	2019	Atezolizumab 1200 mg every 3 weeks	VENTANA SP142 IHC assay	1st line	NSCLC (Non-Squamous)	Platinum based combination therapy	555
OAK	2017	Atezolizumab 1200 mg every 3 weeks	VENTANA SP142 IHC assay	2nd line	NSCLC	Docetaxel	850
POPLAR	2016	Atezolizumab 1200 mg every 3 weeks	VENTANA SP142 IHC assay	2nd line	NSCLC	Docetaxel	287
Javelin 200 Lung	2018	Avelumab 10mg /kg evey 2 weeks	PD-L1 IHC 73-10 pharmDx assay	2nd line	NSCLC	Docetaxel	792
Keynote-006	2015	Pembrolizumab 10 mg/kg every 3 weeks (1.2)	PD-L1 IHC 22C3 pharmDx assay	1st line	Metastatic melanoma	Ipilimumab	834
CheckMate-067	2018	Nivolumab 3 mg/kg every 2 weeks	Rabbit 28-8 (Dako)	1st line	Metastatic Melanoma	Ipilimumab	631
Keynote-048	2018	Pembrolizumab 200 mg/kg every 3 weeks	PD-L1 IHC 22C3 pharmDx assay	1st line	Metastatic HNSCC	Chemotherapy+ cetuximab	882
CheckMate-141	2018	Nivolumab 3 mg/kg every 2 weeks	Rabbit 28-8 (Dako)	1st line	Metastatic HNSCC	Single agent (methotrexate, docetaxel or cetuximab)	361
Keynote-045	2017	Pembrolizumab 200 mg every 3 weeks	PD-L1 IHC 22C3 pharmDx assay)	2nd line	Metastatic Urothelial cancer	Chemotherapy (docetaxel, paclitaxel or vinflunine)	542
IMvigor211	2019	Atezolizumab 1200 mg every 3 weeks.	VENTANA SP142 IHC assay	2nd line	Metastatic Urothelial cancer	Chemotherapy (docetaxel, paclitaxel or vinflunine)	931

NSCLC, non-small cell lung cancer; HNSCC, head and neck squamous cell carcinoma. 1.1,: the third arm, where the pembrolizumab dose was 10mg/kg every 3 weeks was not included, due to the significant difference in dosing, compared to the standard regimen. 1.2: The third arm, where the pembrolizumab dose was 10 mg/kg every 2 weeks was not included, compared to the significant difference in dosing interval compared to the standard regimen.

**Table 2:** Efficacy results between different randomized controlled trials

		Population^1^	HR
**NSCLC 1st line**			
Keynote-042 vs. CheckMate-026	Pembrolizumab vs Nivolumab	PDL-1≥1%	0.81 (0.63-1.05)
Keynote-042 vs. Impower-110	Pembrolizumab vs. Atezolizumab	PDL-1≥1%	1.04 (0.79-1.38)
CheckMate-026 vs. Impower-110	Nivolumab vs. Atezolizumab	PDL-1≥1%	1.29 (0.92-1.79)
Keynote-042 + Kenote-024 vs. CheckMate-026	Pembrolizumab vs Nivolumab	PDL-1≥50%	0.74 (0.50-1.11)
Keynote-042 + Kenote-024 vs Impower-110	Pembrolizumab vs. Atezolizumab	PDL-1≥50%	1.13 (0.73-1.76)
CheckMate-026 vs Impower-110	Nivolumab vs. Atezolizumab	PDL-1≥50%	1.52 (0.89-2.60)
**NSCLC 2nd line**			
Keynote-010 vs CheckMate-017 + CheckMate-057	pembrolizumab vs Nivolumab	PDL-1≥1%	1.01 (0.82-1.25)
Keynote-010 vs OAK + POPLAR	Pembrolizumab vs. atezolizumab	PDL-1≥1%	1.00 (0.78-1.29)
Keynote-010 vs Javelin lung 200	Pembrolizumab vs. avelumab	PDL-1≥1%	0.77 (0.61-0.96)
Checkmate-017 + Checkmate-057 vs OAK + POPLAR NSCLC 2nd line	Nivolumab vs. atezolizumab	PDL-1≥1%	0.99 (0.76-1.28)
CheckMate-017 + Checkmate-057 vs Javelin Lung 200 line PDL1>1%	Nivolumab vs. avelumab	PDL-1≥1%	0.76 (0.58-0.98)
OAK + POPLAR vs Javelin Lung 200	Atezolizumab vs. avelumab	PDL-1≥1%	0.77 (0.58-1.01)
Keynote-010 vs Checkmate-017 + Checkmate-057	Pembrolizumab vs. nivolumab	PDL-1≥50%	1.32 (0.81-2.16)
Keynote-010 vs OAK + POPLAR	Pembrolizumab vs. atezolizumab	PDL-1≥50%	1.23 (0.78-1.94)
Keynote-010 vs Javelin Lung 200 NSCLC PDL1>50 %	Pembrolizumab vs. avelumab	PDL-1≥50%	0.79 (0.55-1.13)
Checkmate-017 + Checkmate-057 vs. OAK + POPLAR	Nivolumab vs. atezolizumab	PDL-1≥50%	0.93 (0.52-1.76)
Checkmate-017 + Checkmate-057 vs. Javelin Lung 200	Nivolumab vs. avelumab	PDL-1≥50%	0.60 (0.36-0.99)
OAK + POPLAR vs Javelin Lung 200	Atezolizumab vs. avelumab	PDL-1≥50%	0.64 (0.40-1.03)
Checkmate-017 + Checkmate-057 vs OAK + POPLAR	Nivolumab vs. atezolizumab	Unselected for PDL-1	0.86 (0.62-1.19)
**Metastatic melanoma 1^st^ line**			
Keynote-006 vs Checkmate-067 Total population	Pembrolizumab vs. nivolumab	Unselected for PDL-1	1.16 (0.89-1.51)
Keynote-006 vs Checkmate-067	Pembrolizumab vs. nivolumab	PDL-1<1%	1.32 (0.67-2.59)
Keynote-006 vs Checkate-067	Pembrolizumab vs. nivolumab	PDL-1≥1%	1.09 (0.71-1.67)
**Metastatic urothelial ca 2^nd^ line**			
Keynote-045 vs Imvigor211	Pembrolizumab vs. atezolizumab	Unselected for PDL-1	0.89 (0.69-1.15)
Keynote-045 vs Imvigor211	Pembrolizumab vs. atezolizumab	PDL-1≥1%	0.70 (0.47-1.05)
**Metastatic HNSCC 1^st^ line**			
Keynote-048 vs Checkmate-141	Pembrolizumab vs. nivolumab	Unselected for PDL-1	1.18 (0.83-1.68)
Keynote-048 vs Checkmate-141	Pembrolizumab vs. nivolumab	PDL-1≥1%	1.25 (0.76-2.06)

^1^ PDL1 status based on the specific assay used in the respective trials. HNSCC, head and neck squamous cell carcinoma.

**Table 3:** Efficacy results between different anti-PD-1/PD-L1 antibodies

	Pembrolizumab	Nivolumab	Atezolizumab	Avelumab
Pembrolizumab		1.02 (0.91-1.14)	0.97 (0.85-1.10)	0.77 (0.64-0.94)
Nivolumab	0.98 (0.88-1.1)		1.05 (0.90-1.23)	0.72 (0.57-0.91)
Atezolizumab	1.03 (0.90-1.17)	0.95 (0.81-1.11)		0.73 (0.58-0.93)
Avelumab	1.3 (1.06-1.56)	1.39 (1.1-1.75)	1.36 (1.07-1.70)	

## Abbreviations

AEs: adverse events
CI: confidence intervals
CTLA-4: anti-cytotoxic-T-lymphocytes-associated protein 4
HR: hazard ratio
ICIs: immune checkpoint inhibitors
IRAEs: immune-related adverse events
NSCLC: non-small cell lung cancer
OR: odds ratio
ORR: objective response rate
OS: overall survival
PD-1: programed death receptor-1
PDL-1: programed death ligand-1
PFS: progression-free survival
RCTs: randomized controlled trials
SAEs: serious adverse events
SE: standard error
