# Supplementary material for: Network Meta-analysis Comparing Efficacy, Safety and Tolerability of Anti-PD-1/PD-L1 Antibodies in Solid Cancers
Source: J Cancer. 2021 May 19;12(14):4372–8. doi: 10.7150/jca.57413 (PMC8176414; doi:10.7150/jca.57413)
Supplement: Supplementary file 1 — Supplementary table S1. [file jcav12p4372s1.pdf]

Study name	CheckMate-026			
	Nivolumab (267)		Chemotherapy (263)	
Events/Grade	Any grade	grade 3 or 4	Any grade	grade 3 or 4
Any event	190 (71%)	47 (18%)	243 (92%)	133 (51%)
Any serious event	46 (17%)	35 (13%)	48 (18%)	41 (16%)
Any event leading to discontinuation of therapy	26 (10%)	21 (8%)	35 (13%)	17 (6%)

Study name	Keynote-024			
	Pembrolizumab (154)		Chemotherapy (150)	
Events/Grade	Any grade	Grade 3 or 4	Any grade	Grade 3 or 4
Any event	113 (73.4%)	41 (26.6%)	135 (90%)	80 (53.3%)
Any serious event	33 (21.4%)	29 (18.8%)	31 (20.7%)	29 (19.3%)
Any event leading to discontinuation of therapy	11 (7.1%)	8 (5.2%)	16 (10.7%)	9 (6%)
Led to death	1 (0.6%)	1 (0.6%)	3 (2%)	3 (2%)

Study name	Keynote-042			
	Pembrolizumab (636)		Chemotherapy (615)	
Events/Grade	Any grade	Grade 3 or 4	Any grade	Grade 3 or 4
Any event	399 (63%)	113 (18%)	553 (90%)	252 (41%)
Any event leading to discontinuation of therapy	57 (9%)	48 (8%)	58 (9%)	43 (7%)
Led to death	13 (2%)	13 (2%)	14 (2%)	14 (2%)

Study name	Impower-110	
	Atezolizumab (286)	Chemotherapy (263)
Events/Grade	Grade 3 or 4	grade 3 or 4
Any event	37 (12.9%)	116 (44.1%)
Any event leading to discontinuation of therapy	18(6.3%)	43 (16.3%)
Led to death	11 (3.8)	11 (4.2%)

Study name	CheckMate-057			
	Nivolumab (287)		Docetaxel (268)	
Events/Grade	Any grade	grade 3 or 4	Any grade	grade 3 or 4
Any event	280 (98%)	132 (46%	265 (99%)	180 (67%)
Any serious event	21 (7%)	15 (5%)	53 (20%)	48 (18%)
Any event leading to discontinuation of therapy	14 (5%)	11 (4%)	40 (15%)	18 (7%)
Led to death	1 (0.3%)		0	

Supplementary appendix

Study name	CheckMate-017			
	Nivolumab (131)		Docetaxel (129)	
Events/Grade	Any grade	grade 3 or 4	Any grade	grade 3 or 4
Any event	76 (58%)	9 (7%)	111 (86%)	71 (55%)
Any serious event	9 (7%)	3 (2%)	31 (24%)	25 (19)
Any event leading to discontinuation of therapy	4 (3%)	2 (2%)	13 (10%)	8 (6%)
Led to death	0		3 (2.3%)	

Study name	Keynote-010			
	Pembrolizumab (339)		Docetaxel (309)	
Events/Grade	Any grade	Grade 3 or 4	Any grade	Grade 3 or 4
Any event	215 (63%)	43 (13%)	251 (81%)	109 (35%)

Study name	OAK trial			
	Atezolizumab (609)		Docetaxel (578)	
Events/Grade	Any grade	Grade 3 or 4	Any grade	Grade 3 or 4
Any event	573 (94%)	227 (37%)	555 (96%)	310 (54%)
Any serious event	194 (32%)	N/A	181 (31%)	N/A
Any event leading to discontinuation of therapy	46 (8%)	46 (8%)	108 (19%)	108 (19%)
Led to death	10 (2%)	10 (2%)	14 (2%)	14 (2%)

Study name	POPLAR trial			
	Atezolizumab (142)		Docetaxel (135)	
Events/Grade	Any grade	grade 3 or 4	Any grade	grade 3 or 4
Any event	136 (96%)	63 (44%)	130 (96%)	76 (56%)
Any serious event	50 (35%)		46 (34%)	
Any event leading to discontinuation of therapy	11 (8%)		30 (22%)	
Led to death	6 (4.2%)		5 (3.7%)	

Study name	Javelin Lung 200			
	Avelumab (393)		Docetaxel (365)	
Events/Grade	Any grade	grade 3,4 or 5	Any grade	grade 3,4 or 5
Any event	251 (64%)	39 (10%)	313 (86%)	180 (49%)

Study name	Keynote-006			
	Pembrolizumab (278)		Ipilimumab (256)	
Events/Grade	Any grade	Grade 3,4 or 5	Any grade	Grade 3,4 or 5
Any event	202 (72.9%)	28 (10.1%)	187 (73%)	51 (19.9)

Study name	CheckMate-067			
	Nivolumab (313)		Ipilimumab (311)	
Events/Grade	Any grade	Grade 3 or 4	Any grade	Grade3 or 4
Any event	270 (86%)	67 (21%)	268 (86%)	86 (28%)
Any event leading to discontinuation of therapy	37 (12%)	24 (8%)	49 (16%)	43 (14%)

Study name	Keynote-048			
	Pembrolizumab (300)		Chemotherapy (287) (cetuximab, platinum and 5-FU)	
Events/Grade	Any grade	Grade 3 or 4	Any grade	Grade 3 or 4
Any event	N/A	164 (54%)	N/A	239 (83%)
Led to death	3 (1%)		8 (2.8%)	

Study name	CheckMate-141			
	Nivolumab (236)		Standard Rx (111) MTX, docetaxel or cetuximab	
Events/Grade	Any grade	Grade 3 or 4	Any grade	grade 3 or 4
Any event	139 (58.9%)	31 (13.1%)	89 (80%)	39 (35%)

Study name	Keynote-045			
	Pembrolizumab (266)		Chemotherapy (255) docetaxel, paclitaxel or vinflunine	
Events/Grade	Any grade	Grade 3 or 4	Any grade	Grade 3 or 4
Any event	162 (60.9%)	40 (15%)	230 (90.2%)	126 (49%)
Any event leading to discontinuation of therapy	15 (5.6 %)	12 (4.5%)	28 (11%)	16 (6.3%)
Led to death	4 (1.5%)	4 (1.5%)	4 (1.6%)	4 (1.6%)

Study name	Imvigor-211	
	Atezolizumab (459)	Chemotherapy (443) docetaxel, paclitaxel or vinflunine
Events/Grade	Any grade	Any grade
Any event	319 (69%)	395 (89%)
